# The role of DNA methylation in the maintenance of phenotypic variation induced by grafting chimerism in *Brassica*

**DOI:** 10.1093/hr/uhad008

**Published:** 2023-01-30

**Authors:** Ke Liu, Tingjin Wang, Duohong Xiao, Bin Liu, Yang Yang, Kexin Xu, Zhenyu Qi, Yan Wang, Junxing Li, Xun Xiang, Lu Yuan, Liping Chen

**Affiliations:** Department of Horticulture, College of Agriculture and Biotechnology, Zhejiang University, Hangzhou 310058, China; Department of Horticulture, College of Agriculture and Biotechnology, Zhejiang University, Hangzhou 310058, China; Department of Horticulture, College of Agriculture and Biotechnology, Zhejiang University, Hangzhou 310058, China; Department of Horticulture, College of Agriculture and Biotechnology, Zhejiang University, Hangzhou 310058, China; Department of Horticulture, College of Agriculture and Biotechnology, Zhejiang University, Hangzhou 310058, China; Department of Horticulture, College of Agriculture and Biotechnology, Zhejiang University, Hangzhou 310058, China; Agricultural Experiment Station, Zhejiang University, Hangzhou 310058, China; Department of Horticulture, College of Agriculture and Biotechnology, Zhejiang University, Hangzhou 310058, China; Department of Horticulture, College of Agriculture and Biotechnology, Zhejiang University, Hangzhou 310058, China; Department of Horticulture, College of Agriculture and Biotechnology, Zhejiang University, Hangzhou 310058, China; Department of Horticulture, College of Agriculture and Biotechnology, Zhejiang University, Hangzhou 310058, China; Department of Horticulture, College of Agriculture and Biotechnology, Zhejiang University, Hangzhou 310058, China

## Abstract

Grafting facilitates the interaction between heterologous cells with different genomes, resulting in abundant phenotypic variation, which provides opportunities for crop improvement. However, how grafting-induced variation occurs and is transmitted to progeny remains elusive. A graft chimera, especially a periclinal chimera, which has genetically distinct cell layers throughout the plant, is an excellent model to probe the molecular mechanisms of grafting-induced variation maintenance. Here we regenerated a plant from the T-cell layer of a periclinal chimera, TCC (where the apical meristem was artificially divided into three cell layers – from outside to inside, L1, L2, and L3; T = Tuber mustard, C = red Cabbage), named rTTT0 (r = regenerated). Compared with the control (rsTTT, s = self-grafted), rTTT0 had multiple phenotypic variations, especially leaf shape variation, which could be maintained in sexual progeny. Transcriptomes were analyzed and 58 phenotypic variation-associated genes were identified. Whole-genome bisulfite sequencing analyses revealed that the methylome of rTTT0 was changed, and the CG methylation level was significantly increased by 8.74%. In rTTT0, the coding gene bodies are hypermethylated in the CG context, while their promoter regions are hypomethylated in the non-CG context. DNA methylation changes in the leaf shape variation-associated coding genes, *ARF10*, *IAA20*, *ROF1*, and *TPR2*, were maintained for five generations of rTTT0. Interestingly, grafting chimerism also affected transcription of the microRNA gene (*MIR*), among which the DNA methylation levels of the promoters of three *MIR*s associated with leaf shape variation were changed in rTTT0, and the DNA methylation modification of *MIR319* was maintained to the fifth generation of selfed progeny of rTTT0 (rTTT5). These findings demonstrate that DNA methylation of coding and non-coding genes plays an important role in heterologous cell interaction-induced variation formation and its transgenerational inheritance.

## Introduction

Grafting is an important agricultural technique that artificially separates, fixes, and combines the tissues or organs of different donors to form plants with different genetic backgrounds [1]. During grafting, the morphological structure and physiological function of plants change, and this is known as grafting-induced phenotypic variation [2]. In agriculture, this phenotypic variation is an important means of improving crops that can rapidly boost yield, improve quality, and enhance resistance to abiotic and biotic stresses [3]. Moreover, in nature, grafting-induced phenotypic variation may be the source of evolutionary innovation [1].

New plasmodesmata are formed between heterologous cells at the graft junction, and a channel for short-distance movement of substances is established [1]. Substances that communicate between heterologous cells can take non-cell-autonomous actions in destination cells, directly or indirectly regulate a plant’s growth and development, then induce new phenotypic characteristics [4]. Lewsey *et al*. [5] found that DNA methylation can be mediated by mobile small RNAs in grafts. In fruit trees heterologous rootstocks can induce genome-wide DNA methylation variation in scions, thereby imparting resistance [6, 7]. In vegetables, after grafting with different rootstocks, the eggplant scion undergoes demethylation, which induces changes in vigor [8]. Thus, grafting-induced DNA methylation changes are closely associated with the formation of plant phenotypic variation.

DNA methylation occurs in three different ways in plants, including CG and CHG (H = A, T, or G) methylation, as well as CHH methylation [9]. DNA methylation can regulate genes at the transcriptional level, including coding genes and non-coding genes such as microRNA genes (*MIR*s) [10, 11]. In different regions of the gene sequence, DNA methylation has different regulatory effects: in promoter regions, DNA methylation typically inhibits gene transcription, while the opposite is also possible; in the gene body region, CG methylation represses or activates gene expression under normal conditions, whereas non-CG methylation plays a major role in gene repression; in introns of several genes, DNA methylation promotes the accumulation of full-length transcripts [12]. CG methylation and CHG methylation are respectively catalyzed by the DNA methylases METHYLTRANSFERASE1 (MET1) and CHROMOMETHYLASE2/3 (CMT2/3), while CHH methylation is mainly maintained by DOMAINS REARRANGED METHYLTRANSFERASE1/2 (DRM1/2) through the small RNA-directed DNA methylation (RdDM) pathway [12]. Interestingly, grafting-induced phenotypic variation is maintained after leaving the graft [13, 14]. Although the cytoplasmic threads of small RNA communication between heterologous cells are blocked, grafting-induced DNA methylation changes to the genome can be preserved [15]. However, whether and how DNA methylation changes caused by cell-to-cell interaction continue to regulate grafting-induced phenotypic variation in progeny are unclear.

A graft chimera is an ideal tool to study the mechanism of grafting-induced phenotypic variation maintenance. Periclinal chimeras are the most stable type, with cells from different donors in the organ that develops from the shoot apical meristem (SAM) [16]. Similar to the graft junction of general grafts, in periclinal chimeras there are extensive short-distance interactions between heterologous cells in organs developed from the SAM [1], providing the basis for grafting-induced phenotypic variation. Importantly, one cell layer in the periclinal chimera can be induced to form new plants, and material exchange between heterologous cells is inhibited, providing an opportunity to analyze the mechanism of grafting-induced variation maintenance in progeny.

**Figure 1:**
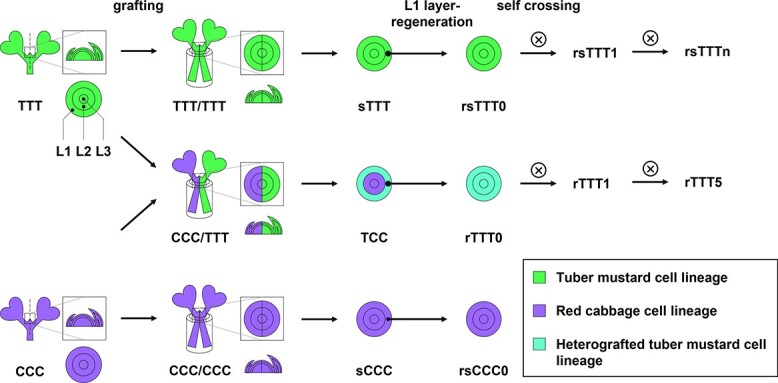
Schematic diagrams of artificial synthesis of tuber mustard (*B. juncea*) and red cabbage (*B. oleracea*) grafting chimera TCC and production of its progeny. Periclinal chimera TCC (L1-L2-L3, T = Tuber mustard cells, C = red Cabbage cells) was generated by *in vitro* grafting between tuber mustard (*B. juncea*, TTT) and red cabbage (*B. oleracea*, CCC). The tuber mustard cell lineage located in the epidermis (L1 layer) of leaf axils of TCC was regenerated into plantlets, named rTTT0 (r = regenerated), and the sexual progeny of rTTT0 (rTTT1 to rTTT5) were produced by self-crossing. Controls (sTTT and sCCC, s = self-grafted) were obtained by self-grafting of tuber mustard and red cabbage, respectively, and the L1-layer-regenerated plants were induced by the same method of inducing rTTT0, and were named rsTTT0 and rsCCC0. The control sexual progeny produced by self-crossing of rsTTT0 were named rsTTTn (*n* = 1–5).

In this study, a periclinal chimera (TCC) synthesized by the grafting of tuber mustard (*Brassica juncea*, TTT) and red cabbage (*Brassica oleracea*, CCC) was used as the material, and the T-cell layer of the chimeras was induced to regenerate plants (rTTT0) (Fig. 1). Compared with the control (rsTTT0), rTTT0 had grafting chimerism-induced phenotypic variation in leaf, stem, and flower development. The leaf shape variation induced by grafting chimerism could be maintained in progeny. Transcriptome analysis of rTTT0 and rsTTT0 was performed to identify genes that regulate these phenotypic variations. Furthermore, the methylomes of rTTT0 and rsTTT0 were comparatively analyzed, and the genome-wide methylation of rTTT0 was significantly changed. Differentially methylated regions (DMRs) were identified in the promoters and gene bodies of coding genes and *MIR*s associated with leaf shape development. DNA methylation variation in the promoters of *TPR2* and *MIR319* could be stably inherited in sexual progeny of rTTT0. The results of this study shed light on the transgenerational inheritance of beneficial heterologous cell interaction-induced traits mediated by DNA methylation.

## Results

### Grafting chimerism induces abundant phenotypic variation

To explore the effects of grafting on phenotypic variation, a periclinal chimera of tuber mustard (*B. juncea*) and red cabbage (*B. oleracea*) was synthesized by *in vitro* grafting. In a previous study, we identified the distribution of cells of different species in the SAM by *in situ* hybridization, and found that the T (T = Tuber mustard) cell lineage was located in the L1 layer, while the C (C = red Cabbage) cell lineage was located in the L2 and L3 layers [17]. Based on the order of L1-L2-L3, the periclinal chimera was TCC. Tuber mustard plants were then regenerated (rTTT0, r = regenerated) from epidermal cells (L1 layer cells) at leaf axils of the stem segments of the chimera TCC. rTTT0 was identified by a specific molecular marker (*atpA*) that can distinguish tuber mustard and red cabbage cells, and it was found that the PCR amplification product of rTTT0 was consistent with TTT (Supplementary Data Fig. S1), indicating that there were no CCC-derived cells in rTTT0. The control was regenerated from the epidermis of the leaf axils of the stem segment of the self-grafted tuber mustard using the same treatment, and named rsTTT0 (s = self-grafted) (Fig. 1).

**Figure 2:**
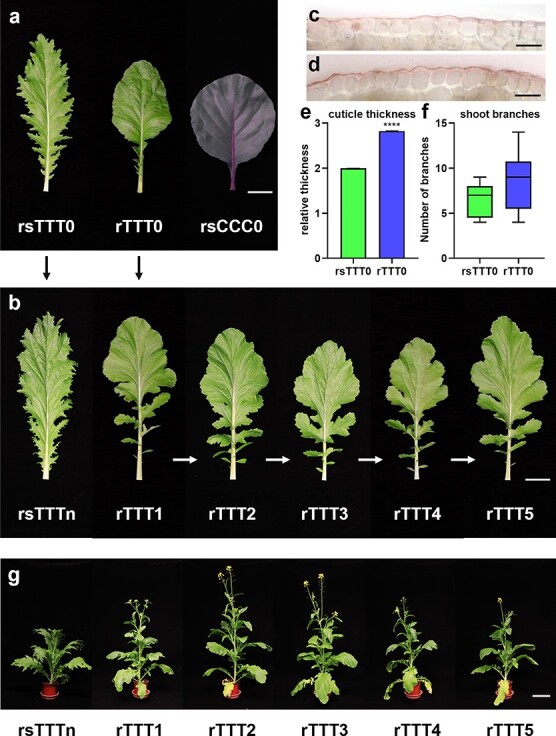
Grafting chimerism induces abundant phenotypic variation in tuber mustard. **a**, **b** Leaf morphologies of rsTTT0, rTTT0, and rsCCC0 (**a**) and sexual progeny of rsTTT0 and rTTT0 (**b**). Scale bars, 5 cm. Self-crossing is indicated by arrows. **c**, **d** Light micrographs of sections of epidermal cells of rTTT0 (**c**) and rsTTT0 (**d**), showing Oil Red O staining. Scale bars, 100 μm. **e** Relative thickness of cuticle in rsTTT0 and rTTT0. Data represent mean ± standard deviation. Asterisks indicate significant differences between rTTT0 and rsTTT0 by analysis of two-tailed unpaired Student’s *t*-tests. ^****^P <0 .0001. **f** Numbers of primary cauline branches in rsTTT0 and rTTT0. **g** First flowers of rTTT0 sexually selfed progeny generally open earlier. Scale bars, 15 cm.

Phenotypic observation revealed that rTTT0 exhibited a variety of phenotypic variation in different organs. The leaf shape of rTTT0 changed (Fig. 2a), resulting in reductions in the aspect ratio (27.94%) and leaf area (24.21%) (Supplementary Data Fig. S2a–c). rTTT0 had fewer leaflets at the base of the leaf and shallower serrations than the control’s leaves, resulting in a leaf morphology resembling that of red cabbage (Fig. 2a). Notably, the leaf margin variation was stably maintained in the first to fifth generations (rTTT1 to rTTT5) of rTTT0 selfed progeny (Fig. 2b). In addition, the cuticle thickness of rTTT0 epidermal cells was significantly increased (Fig. 2c and d, Supplementary Data Fig. S2d–f). Staining with Oil Red showed that the cuticle of rTTT0’s epidermal cells was 1.41 times thicker than that of rsTTT0 (Fig. 2e). In shoots there were more branches on the stem of rTTT0 than on the stem of rsTTT0 (Fig. 2f), although there were differences in the numbers of branches among different individuals (Fig. 2f).

Grafting chimerism affects the development of organ morphology, and changes the developmental transition from vegetative to reproductive. Compared with rsTTT0, the bolting time of rTTT0 was ~4 days earlier (Supplementary Data Fig. S2g). Observing the flowering time of the first to fifth generations (rTTT1 to rTTT5) of the rTTT0 selfed progeny and the control rsTTTn (*n* = 1–5), the flowering time of the progeny was earlier than that of the control (Fig. 2g). Thus, these results indicate that grafting chimerism induces abundant phenotypic variation in plant growth and development, and some phenotypic variation can be maintained in the progeny.

### Grafting chimerism alters expression of development-related coding genes

**Figure 3:**
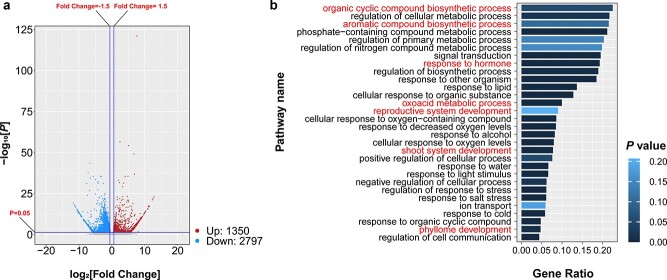
Coding gene expression profiles and pathway changes in tuber mustard after grafting chimerism. **a** Volcano plot showing DEGs in rTTT0 and rsTTT0. Significant values (fold change >1.5 and *P* <0 .05) are marked in blue or red for downregulated and upregulated coding genes in rTTT0. The *P* values were calculated by the hypergeometric probability test and adjusted by the Benjamini–Hochberg method. **b** GO term enrichments for DEGs in rTTT0 and rsTTT0. The 30 terms with the largest gene ratios are plotted in order of gene ratio. The *P* values were adjusted by the Benjamini–Hochberg method. Pathway names associated with grafting-induced phenotypic variations are highlighted in red.

To identify genes involved in grafting chimerism-induced phenotypic variation, RNA-Seq was used to analyze the coding gene expression of rTTT0 and rsTTT0. In the analysis of rTTT0 and rsTTT0 transcriptomes, 4147 differentially expressed coding genes (DEGs) were identified. Compared with rsTTT0, 2797 genes were downregulated in rTTT0, which was 2.07 times the number of upregulated genes (1350) (Fig. 3a).

Gene ontology (GO) enrichment analysis of the DEGs indicated that they were mainly enriched in organic cyclic compound biosynthetic process, the regulation of cellular metabolic process, and aromatic compound biosynthetic process (Fig. 3b). To identify the pathways associated with phenotypic variation, the GO categories were analyzed. Two pathways were associated with leaf development, response to hormone, and phyllome development (Fig. 3b). The pathways associated with cuticle formation are organic cyclic compound biosynthetic process, aromatic compound biosynthetic process, and oxoacid metabolic process (Fig. 3b). In addition, two pathways were associated with stem development and developmental phase transition (Fig. 3b). Next, we performed Kyoto Encyclopedia of Genes and Genomes (KEGG) enrichment analysis of the DEGs and found that they were specifically enriched in 30 KEGG categories, including brassinosteroid biosynthesis (ko00905), cutin, suberin, and wax biosynthesis (ko00073), and zeatin biosynthesis (ko00908) (Supplementary Data Fig. S3).

**Figure 4:**
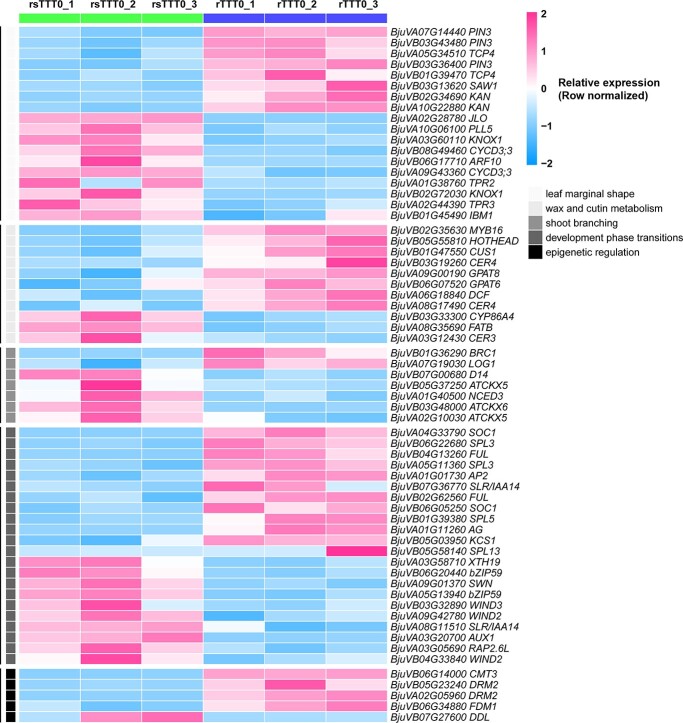
Key DEGs between rTTT0 and rsTTT0 regulated grafting chimerism-induced phenotypic variation. Heat map showing the expression of grafting chimerism-induced phenotypic variation-related DEGs between rTTT0 and rsTTT0. *P* <0 .05, fold change >1.5, *n* = 3 samples. The *P* value obtained based on the negative binomial distribution model was adjusted using the Benjamini–Hochberg method. The color scale on right shows the normalized expression values from 2 (red) to −2 (blue).

Furthermore, a detailed analysis of DEGs and enrichment results identified 58 key coding genes that were involved in grafting chimerism-induced phenotypic variation. It was found that there were 18 key genes involved in leaf shape variation, including *TCP4* (*BjuVA05G34510*, *BjuVB01G39470*), *KAN* (*BjuVA10G22880*, *BjuVB02G34690*), *KNOX1* (*BjuVA03G60110*, *BjuVB02G72030*), *ARF10* (*BjuVB06G17710*), and *TPR2* (*BjuVA01G38760*) (Fig. 4). In addition, 11 genes were identified that were involved in cuticle formation, including *CER4* (*BjuVB03G19260*, *BjuVA08G17490*) and *MYB16* (*BjuVB02G35630*) (Fig. 4). Notably, there were significant changes in the expression levels of five DNA epigenetic regulators in rTTT0, including the DNA methyltransferases *DRM2* (*BjuVB05G23240*, *BjuVA02G05960*) and *CMT3* (*BjuVB06G14000*) (Fig. 4). Thus, these results suggest that grafting chimerism affects the transcription of development-related coding genes, and upregulation of *DRM2* and *CMT3* may affect genome-wide changes in DNA methylation levels.

### Grafting chimerism induces changes in genome-wide DNA methylation levels

**Figure 5:**
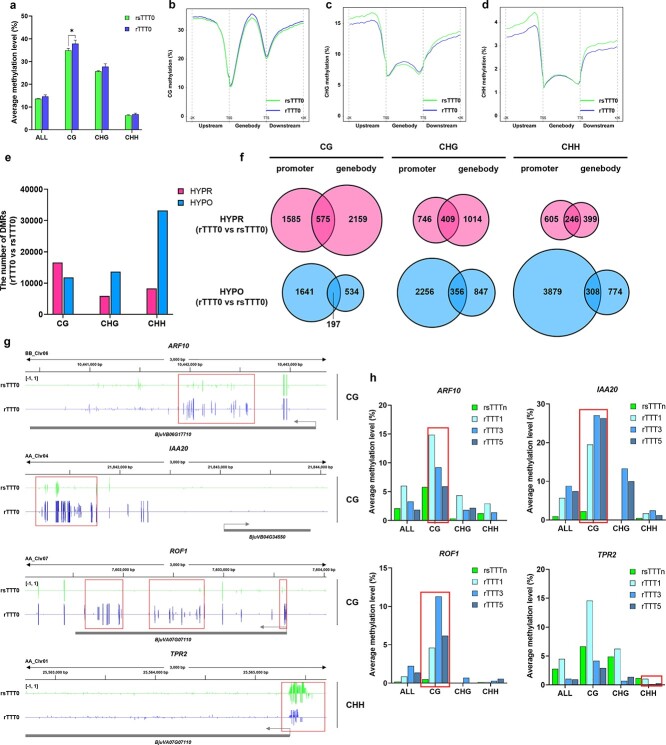
Grafting chimerism-induced changes in DNA methylation levels of coding genes associated with phenotypic variation. **a** Average levels of all DNA methylation, CG methylation, CHG methylation, and CHH methylation in rsTTT0 and rTTT0. Asterisks indicate significant differences between rTTT0 and rsTTT0 by analysis using the two-tailed unpaired Student’s *t*-test. ^*^P <0 .1. *n* = 3 biologically independent samples. **b**–**d** Changes in CG (**b**), CHG (**c**), and CHH (**d**) methylation in coding gene regions and their flanking regions of 2 kb (upstream and downstream of coding genes) in rsTTT0 and rTTT0. *n* = 3 biologically independent samples. **e** Numbers of hyper-DMRs and hypo-DMRs identified in rTTT0 relative to rsTTT0. DMRs were independently identified for CG, CHG, and CHH sites. HYPR, hypermethylated; HYPO, hypomethylated. **f** Venn diagrams showing DMR-associated coding genes with hypermethylation and hypomethylation of CG, CHG and CHH contexts in the promoter region and gene body. Overlaps represent DMGs containing DMRs in both gene body and promoter regions. **g** Genome-browser views of DNA methylation levels in CG contexts of *ARF10*, IAA20, *ROF1*, and DNA methylation levels in CHH contexts of *TPR2* in rTTT0 and rsTTT0. The vertical bars on each track show the DNA methylation levels. The regions in the red box are the DMRs of rTTT0 and rsTTT0. *n* = 3 biologically independent samples. **h** Changes in DNA methylation levels of *ARF10*, *IAA20*, *ROF1*, and *TPR2* in the first, third, and fifth sexual progeny of rTTT0 selfing (rTTT1, rTTT3, rTTT5) and controls (rsTTTn), respectively. The red box indicates the maintenance of DNA methylation variation of *ARF10*, *IAA20*, *ROF1*, and *TPR2* in sexual progeny.

To investigate whether upregulation of DNA methyltransferases would lead to methylation changes after grafting chimerism, genome-wide methylation analysis of rTTT0 and rsTTT0 was performed using whole-genome bisulfite sequencing. Each methylome was sequenced with >15-fold coverage, and >72% of the tuber mustard genomic cytosine positions were covered in each sample. The global DNA methylation levels in all three contexts were markedly increased, especially in the CG (Fig. 5a). To acquire an overview of the DNA methylation profiles of the coding genes, DNA methylation levels in the gene body region and their adjacent regions (2 kb region upstream of the gene transcription start site and 2 kb region downstream of the gene transcription termination site) in rTTT0 and rsTTT0 were compared. Increased levels of DNA methylation in the CG were observed in all regions (Fig. 5b), which was consistent with the global changing methylation pattern (Fig. 5a). However, dramatic decreases in non-CG methylation levels were detected in the adjacent regions, especially 2 kb upstream of TSS (Fig. 5b). These results suggest that increased genome-wide DNA methylation level were induced by grafting chimerism, and that this was likely responsible for the transcriptional regulation in rTTT0.

### Grafting chimerism changes the transcription of leaf development-related coding genes through DNA methylation

To study the role of DNA methylation in grafting chimerism-induced gene transcriptional changes, DMRs were screened in rTTT0 and rsTTT0, and a total of 89 476 DMRs were identified. In the CG context, there were more hypermethylated DMRs (16 575) than hypomethylated DMRs (11820) in rTTT0 (Fig. 5e). In contrast, hypomethylation in rTTT0 was observed in the non-CG context (13 698 in CHG and 33 182 in CHH) (Fig. 5e). To assess the effects of DNA methylation on gene expression, the DMR-associated genes (DMGs) were identified in rTTT0. In the CG and CHG context, there were more DMGs with hypermethylated DMRs in their gene body regions than in their promoter regions (Fig. 5f). In contrast, in the CHH context more DMGs had hypermethylated DMRs in their promoter regions (605) than in their gene body regions (399) (Fig. 5f). However, in the hypomethylation DMGs, the numbers of genes with CHH DMRs in their promoter (3879) was much greater than the number with CHH DMRs in their gene body regions (774) (Fig. 5f). This suggests that grafting chimerism may induce phenotypic variation by changing CG methylation levels in protein-coding gene bodies and reducing non-CG methylation levels in their promoters.

To confirm these results, DMGs were compared with the DEGs associated with phenotypic variation and we found that 10 DMGs regulating leaf development were significantly differentially expressed in rTTT0, including *ARF10* (*BjuVB06G17710*, involved in leaf margin serration development), *CYCD3;3* (*BjuVA09G43360*, *BjuVB08G49460*, important for determining cell number in developing lateral organs), *PLL5* (*BjuVA10G06100*, involved in leaf development), *IBM1* (*BjuVB01G45490*, a histone H3K9 demethylase involved in leaf development), *TPR2* (*BjuVA01G38760*, interacts with TIE1 to regulate leaf margin development), *PIN3* (*BjuVB03G36400*, *BjuVB03G43480*, a regulator of auxin efflux and involved in differential growth), *IAA20* (*BjuVA04G34550*, encodes a member of the Aux/IAA family of proteins implicated in auxin signaling), and *ROF1* (*BjuVA07G07110*, has slight effects on PGP1-mediated IAA transport). Furthermore, we associated hypermethylated and hypomethylation DMRs in the gene body and promoters with gene expression levels in rTTT0 and rsTTT0. We found that in rTTT0 the exon region of *ARF10* and *ROF1* was overlapped by a hypermethylated CG DMR with reduced expression levels, the promoter region of *IAA20* had a hypermethylated CG DMR with increased expression level, and the promoter region of *TPR2* had a hypomethylated CHH DMR with reduced expression levels (Fig. 5g).

In our previous study, we found that the maintenance of graft-induced phenotypic variation was related to DNA methylation [14], so we investigated whether the DNA methylation changes induced by grafting chimerism in these coding genes would be maintained in the progeny. Notably, bisulfite sequencing indicated that grafting chimerism-induced changes in DNA methylation levels of *ROF1*, *IAA20*, and *TPR2* could be maintained from the first generation (rTTT1) to the fifth generation (rTTT5) of sexual progeny of rTTT0 (Fig. 5h). Taken together, these results demonstrate that grafting chimerism affects DNA methylation levels of coding genes associated with leaf variation, and that this mechanism is maintained in sexual progeny.

### Grafting chimerism alters expression patterns of non-coding genes

**Figure 6:**
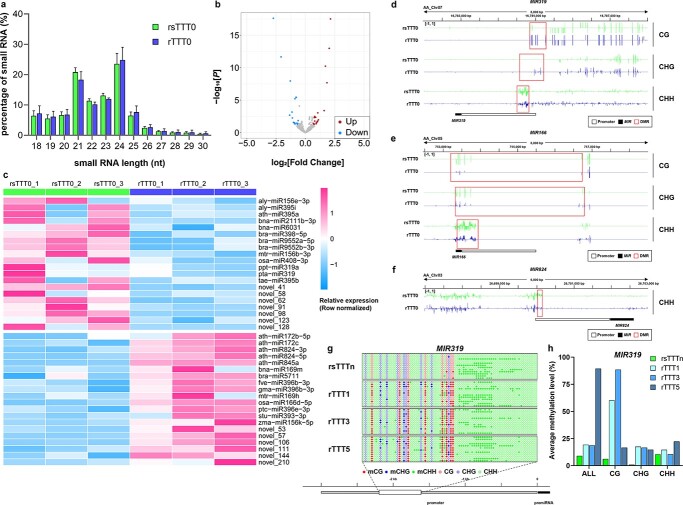
Grafting chimerism affects DNA methylation of the promoter regions of *MIR*s associated with phenotypic variation. **a** Small RNA size distributions in rsTTT0 and rTTT0. Data represent mean ± standard deviation. **b** Volcano plot of miRNA expression changes in rsTTT0 and rTTT0. Significant values (fold change >1.5 and *P* <0 .05) are marked in blue or red for downregulated or upregulated coding genes in rTTT0. The *P* values were calculated using the hypergeometric probability test. **c** Heat map of miRNA expression changes between rsTTT0 and rTTT0. *P* < 0.05, fold change >1.5, *n* = 3 samples. The *P* value was based on the negative binomial distribution model and adjusted using the Benjamini–Hochberg method. The color scale at right shows the normalized expression values. **d**–**f** DNA methylation levels in the CG, CHG, and CHH contexts of the promoter of *MIR319* (**d**) *MIR166* (**e**), and *MIR824* (**f**) are shown with screenshots from the Integrative Genome Browser display of whole-genome bisulfite sequencing data. The vertical bars on each track show the DNA methylation levels. The regions in the red box are the DMRs of rTTT0 and rsTTT0. *n* = 3 biologically independent samples. **g** Analysis of DNA methylation in the promoter regions of *MIR319* in the sexual progeny of rTTT0 and its control (rsTTTn) using individual-locus bisulfite sequencing. The filled circles indicate methylated cytosines and the empty circles indicate unmethylated cytosines. **h** Mean methylation levels in the C, CG, CHG, and CHH contexts of the promoter of *MIR319* in the sexual progeny of rTTT0 and its controls (rsTTTn).

Grafting chimerism induced changes in the genome-wide DNA methylation level in tuber mustard. DNA methylation levels alter the transcription levels of protein-coding genes, and may also affect the transcription of non-coding genes such as small RNAs. To test this hypothesis, differences in the small RNAs of rTTT0 and rsTTT0 were compared. In rTTT0 and rsTTT0 the sizes of small RNAs were mainly distributed between 21 and 24 nucleotides (nt), accounting for 65.36 and 68.84% of all small RNAs, respectively (Fig. 6a). Compared with rsTTT0, the proportion of 21- to 23-nt small RNAs in rTTT0 was lower, and the proportion of 24-nt small RNAs was higher. miRNA is the main component of 21- to 23-nt small RNAs (Fig. 6a). Comparing the miRNA expression levels of rTTT0 and rsTTT0, 20 miRNAs were significantly upregulated and 20 miRNAs were significantly downregulated (Fig. 6b and c).

**Figure 7:**
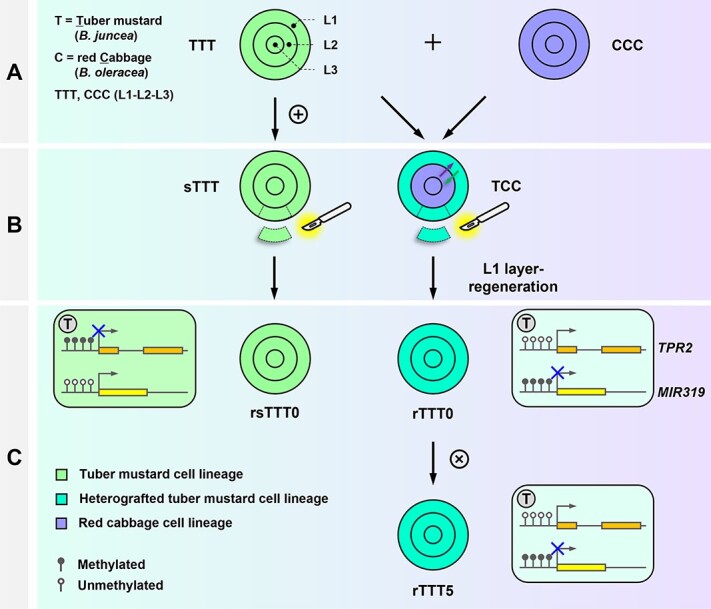
A model of transgenerational inheritance of DNA methylation-mediated grafting chimerism-induced leaf shape variation. Grafting chimerism induces DNA methylation variation in the promoters or gene body regions of protein-coding genes (e.g. *TPR2*) and non-coding genes (*MIR319*), and directly or indirectly regulates the transcription of genes related to leaf shape variation. Grafting chimerism-induced DNA methylation variation can be stably maintained in sexual offspring. A, graft chimera; B, L1 layer regeneration; C, self-crossing.

To investigate whether miRNAs are involved in grafting chimerism-induced phenotypic variation, TargetFinder was used to predict the target genes of miRNAs, and a total of 323 target genes were identified. A total of 160 target genes were post-transcriptionally regulated by 40 differentially expressed miRNAs in rTTT0 (Supplementary Data Table S1). The target gene functions were predicted by BLAST sequence alignment with *Arabidopsis thaliana* genes, and this process indicated that these genes are involved in regulating biological processes such as leaf development, shoot branching, floral organ senescence, seed viability, responses to pathogen, cuticle formation, and cell elongation (Supplementary Data Table S2). Furthermore, miR319, miR166, and miR824 were identified as major regulators of grafting chimerism-induced leaf variation in rTTT0. Notably, miR319 and miR166 were reported to play key roles in leaf morphogenesis [18], and the expression levels of the miR319 target gene *TCP4* were significantly upregulated (Fig. 4). The target gene of miR824, *NAC078* (*BjuVA03G01600*), is a homologous gene of *ANAC078* in *A. thaliana*, which directly regulates leaf margin development [19]. Additionally, we predicted that novel_98 is a key miRNA that regulates cuticle variation, and its target gene, *MYB16* (*BjuVB02G35630*), was significantly upregulated in rTTT0 (Fig. 4). These results suggest that grafting chimerism induced changes in the expression profiles of microRNA genes (*MIR*s), which modulated grafting chimerism-induced phenotypic variation and may be regulated by DNA methylation.

### DNA methylation in *MIR* promoter regions after grafting chimerism is maintained in progeny

To investigate whether transcriptional changes in *MIR*s associated with phenotypic variation are regulated by DNA methylation after grafting chimerism, differences in DNA methylation levels of promoters of key *MIR*s involved in phenotypic variation were assessed in rTTT0, including *MIR319*, *MIR166*, and *MIR824*. In rTTT0, the average DNA methylation level of the *MIR319* promoter was increased, whereas the CHH methylation levels of the *MIR166* and *MIR824* promoter regions were decreased (Fig. 6d–f). The results suggest that variations in DNA methylation levels led to changes in *MIR* expression that regulated phenotypic variation.

To investigate whether the effects of DNA methylation variation on *MIR*s could be maintained in offspring, DNA methylation levels of key *MIR* promoter regions were analyzed in the sexual offspring of rTTT0 (rTTT1, rTTT3, and rTTT5) by bisulfite sequencing PCR (BSP). Increased level of DNA methylation in the promoter of *MIR319* could be maintained in sexual progeny of rTTT0 (Fig. 6g and h). These results suggest that the DNA methylation variation of *MIR* sequences induced by grafting chimerism can be inherited in sexual progeny, which is the key reason for the long-term maintenance of grafting chimerism-induced leaf shape variation.

## Discussion

Grafting can rapidly improve crops [1]. After grafting, the crops exhibit beneficial grafting-induced traits [3]. However, after leaving the grafted state, the mechanism of how the grafting-induced phenotypic variation is maintained during sexual and/or asexual reproduction is unclear. In this study， a multi-organ heterologous cell interaction system was constructed using previously synthesized materials: tuber mustard and red cabbage periclinal chimera [17]. This is an ideal system for studying the maintenance mechanisms of grafting-induced variation because it allows single cell layers to be induced from it for morphological and molecular analysis. Tuber mustard (rTTT0) was regenerated from the L1 cell layer of the grafting chimera (TCC), and its phenotype, coding and non-coding gene expression, and genomic DNA methylation were analyzed. It was found that grafting chimerism induced epigenetic variation in tuber mustard, regulating leaf development at transcriptional and post-transcriptional levels, and this epigenetic regulation mechanism could be transmitted to sexual progeny (Fig. 7).

### Heterologous cell interaction induces genome-wide DNA methylation variation

In general, the donors of rootstocks and scions are from different genotypes, species, genera, or families, and have different genetic backgrounds [3]. After grafting, the cells of rootstock and scion at the junction undergo dedifferentiation and redifferentiation to form new plasmodesmata and vasculature [20]. These channels facilitate cell-to-cell interactions between heterologous cells in grafts. To explore the implications of this communication, we regenerated tuber mustard (rTTT0) from TCC (Fig. 1). Genome-wide DNA methylation analysis of rTTT0 and rsTTT0 indicated that grafting chimerism induced an increase in the genome-wide DNA methylation level of tuber mustard. Particularly, CG methylation was significantly increased by 8.74% (Fig. 5a). In previous studies of annual and perennial plants, it has also been reported that heterologous cell interaction changed the genome-wide methylation profile of scions. After heterografting of *Malus prunifolia* ‘Fupingqiuzi’ and commercial apple (*M. × domestica* ‘Golden Delicious’), the DNA methylation levels in CG and CHG of ‘Golden Delicious’ were significantly increased [6]. Similarly, interspecific heterografting of cucumber (*Cucumis sativus*) and melon (*Cucumis melo*) also resulted in increased DNA methylation in the scion [21].

In heterologous grafts, the microenvironment of cells *in vivo* changes due to differences in the genetic background of adjacent cells. Increasing evidence suggests that plant cells can regulate genomic DNA methylation in response to environmental changes to reduce the impact on genome stability [22]. In this study, the expression of DNA methyltransferases (CMT3 and DMR2) in rTTT0 was significantly upregulated (Fig. 4), which may have been a response of the cells to changes in the microenvironment. Moreover, new plasmodesmata and vasculature were established between heterologous cells in the graft, providing a channel for the exchange of intercellular substances and facilitating cell-to-cell interactions [1, 23]. These substances, such as hormones, proteins, RNA, and DNA, can move into heterologous cells through plasmodesmata and vascular systems and act in a non-cell-autonomous manner, affecting the structure and function of cells [2]. In recent years numerous studies have shown that small RNA can move both long and short distances in plants and mediate DNA methylation in destination cells [24]. For example, in *A. thaliana*, 24-nt small RNAs in shoots were found to move within the graft and mediate DNA methylation in roots [25]. Therefore, the movement of small RNAs in a graft may directly lead to heterologous cell interaction-induced DNA methylation variation.

### DNA methylation directly or indirectly regulates genes involved in grafting-induced phenotypic variation

DNA methylation can regulate gene expression in plants, affecting physiology and development, and inducing phenotypic variation [26, 27]. It can directly regulate coding genes at the transcriptional level, and it can indirectly regulate coding genes by regulating the transcription of microRNAs [10, 11]. In this study, the transcriptomes and miRNomes of rTTT0 and rsTTT0 were identified, and differentially expressed coding genes (4147) and miRNAs (40) were identified. There were 56 coding genes and 11 non-coding genes related to phenotypic variations. DNA methylation levels in the promoters and gene bodies of 10 coding genes related to leaf shape development were changed, including the coding genes *ARF10* (*BjuVB06G17710*), *PIN3* (*BjuVB03G36400*, *BjuVB03G43480*), *IAA20* (*BjuVA04G34550*), and *TPR2* (*BjuVA01G38760*). In rTTT0, the CG methylation level of the exon of *ARF10* was increased, while the CHH methylation level of the promoter of *TPR2* decreased, and their expression levels were downregulated according to the transcriptomic data (Fig. 4). In *A. thaliana*, the gene homologous to *ARF10* is involved in auxin signal transduction and is overexpressed, resulting in an increased depth of serrations of the rosette leaf margin [28]. In this study, heterologous cell interaction increased the DNA methylation level of *ARF10* and suppressed its expression, possibly resulting in the reduced depth of serrations. TPL/TPR corepressors are recruited by TIE to form a complex that inhibits the expression of *TCP* to form serrations in leaves [29]. In rTTT0, it is possible that DNA methylation-mediated low *TPR2* expression affected leaf serration formation. These results suggest that DNA methylation induces phenotypic variation directly by regulating the transcription of coding genes.

Interestingly, DNA methylation levels in the promoter of *MIR*s associated with phenotypic variation also changed. In rTTT0, the DNA methylation level of the promoter of *MIR319* was increased (Fig. 6d), inhibiting its post-transcriptional silencing of the target gene TCP4 (*BjuVA05g34510*) (Fig. 4). Therefore, DNA methylation indirectly regulates the expression of phenotype-related coding genes by regulating the transcription of non-coding genes, resulting in phenotypic variation.

### Transgenerational inheritance of grafting chimerism-induced trait variation depends on the maintenance of DNA methylation variation

To determine whether grafting chimerism-induced phenotypic variation could be transmitted to sexual offspring, we generated sexual offspring of rTTT0 via self-pollination for five generations (rTTT1 to rTTT5). Variation in leaf margins of rTTT0 could be stably maintained in sexual offspring. In plants, DNA methylation plays a key role in transgenerational inheritance [30]. In the present study, DNA methylation variation of leaf shape-related genes in rTTT0 could be stably inherited in sexual offspring (Fig. 5g). Therefore, we speculate that grafting-induced variant traits depend on the maintenance of DNA methylation variation.

In previous studies, we used the synthetic periclinal chimeras TTC and TCC to explore the genetic mechanism of grafting-induced phenotypic variation. Cao *et al*. generated sexual progeny developed from gametes produced in the L2 layer of TTC through selfing [14]. The DNA methylation of TTC sexual progeny were analyzed by MSAP (methylation-sensitive amplified polymorphism), and it was found that the first generation of sexual progeny had 5.29–6.59% DNA methylation variation, and 31.58% of the changes could be stably maintained until the fifth generation of sexual progeny. The changes in methylomes of sexual progeny may be derived from grafting-induced somatic variation in the L2 layer, or may be affected by heterologous cells in the L3 layer when the L2 layer formed gametes. Subsequently, Yu *et al*. induced somatic cells of the L2/L3 layer in TCC to regenerate plants, and found that the regenerated plants had phenotypic variation, and this grafting-induced variation could be maintained in asexual progeny and was regulated by DNA methylation [15]. Therefore, after the somatic cells leave the graft chimera, the grafting-induced phenotypic variation of the regenerated plants is regulated by DNA methylation, and the maintenance of this phenotypic variation in sexual progeny also requires the participation of DNA methylation.

Similarly, it was found that grafting-induced DNA methylation mediated the inheritance of grafting-induced growth vigor enhancement in sexual offspring of *A. thaliana* and tomato (*Solanum lycopersicum*) [31]. The symmetric type of DNA methylation is relatively stable during gamete formation, fertilization, and embryonic development [32–36], while the asymmetric type of DNA methylation is reprogrammed. In this study, two different types of DNA methylation variants were found in leaf shape-related genes, and these variants could be stably inherited in sexual offspring (Figs 5h and 6g and h). However, the mechanism by which DNA methylation is maintained or reprogrammed in the progeny of the graft remains to be further investigated.

Collectively, our results suggest that the formation and transgenerational inheritance of heterologous cell interaction-induced phenotypic variation depend on the regulation of transcriptional levels of coding and non-coding genes via DNA methylation. The study also shows the potential of the graft chimera for future crop improvement, and provides evidence for species evolution.

## Materials and methods

### Plant material

Periclinal chimeras of tuber mustard (*B. juncea* var. *tumida*) and red cabbage (*B. oleracea* var. *capitata*) were generated as previously described [15, 37]. Briefly, 6-day-old seedlings were cut in half, including a cotyledon and most of the SAM. Dissected tissue was immersed in mixed hormone solution, which consisted of 1 mg/l naphthylacetic acid and 2 mg/l 6-benzyl aminopurine (6-BA) for 1 minute, then micrografted with silicone tubes. After 2 weeks of culture in Murashige and Skoog (MS) medium with 0.1 mg/l 6-BA [38], united SAMs were cultured in the same medium to induce chimeric shoots. In accordance with a previously described method, TCC periclinal chimeras (L1-L2-L3) were identified as those with an L1 layer from tuber mustard (TTT) and L2 and L3 layers from red cabbage (CCC) in the SAM. After subculture of TCC in ½ MS for 1 month, stem segments with single axillary buds were cut and inoculated into MS medium containing 0.2 mg/l 6-BA. The axillary buds were excised after a month of culture and then transferred to MS medium containing 0.1 mg/l 6-BA to regenerate shoots from the epidermal cells of the tuber mustard from which the axillary buds were removed. The regenerated shoots (rTTT0) were rooted in ½ MS and moved to a greenhouse, followed by self-crossing for five generations (rTTT1 to rTTT5). The tuber mustard and red cabbage were self-grafted to synthesize sTTT and sCCC, and rsTTT0 and rsCCC0 were regenerated as described above.

### Cell type identification of rTTT0 plants

Genomic DNA was isolated from the candidate rTTT0 plants using the Plant Genomic DNA Kit (TIANGEN). The cell type identification of rTTT0 plants was performed according to Li *et al*. with minor modifications [17]. Briefly, each 20-μl reaction system contained 50 ng of genomic DNA, 500 nM specific primers (*atpA*, listed in Supplementary Data Table S3), and 2 × Green Taq Mix (Vazyme). PCR was performed using the following program: 95°C for 5 minutes followed by 30 cycles of 95°C for 15 seconds, 55°C for 30 seconds, and 72°C for 15 seconds, then 72°C for 5 minutes. The genomic DNA of tuber mustard and red cabbage was amplified into 1050- and 1500-bp fragments, respectively.

### Cuticle staining and thickness analysis of epidermal cells

To analyze grafting chimerism-induced variation in epidermal cuticle thickness, stem sections from rTTT0 and rsTTT0 were collected for hand-sectioning. As previously described, Oil Red was used to stain the stem sections [15]. In brief, the sections were immersed in 60% isopropanol, stained in Oil Red (saturated in 60% isopropanol) for 30 minutes, eluted with isopropanol, then viewed under a microscope with a Plan Fluor 40×/0.75 objective. The cuticle thickness of epidermal cells was analyzed using the cell membrane thickness analysis tool in ImageJ software (https://imagej.net/software/fiji/).

### RNA-Seq library construction and sequencing

Fourth leaves were collected from 30-day-old rTTT0 and rsTTT0 seedlings, and total RNA was extracted from them using TRIzol (Invitrogen). The NEBNext Ultra RNA Library Prep Kit for Illumina was used to construct RNA-Seq libraries, which were then sequenced on the Novaseq 6000 platform to produce 150-bp paired-end reads. The raw reads were filtered to remove low-quality reads and adapter sequences, and then the processed reads from each library were mapped to the tuber mustard reference genome (http://39.100.233.196:82/download_genome/Brassica_Genome_data/Braju_tum_V2.0) using HISAT2 (v.2.0.5). The abundance of mRNAs from each gene was identified and standardized to counts per million reads (c.p.m.) using the subread program. The ‘DESeq2’ package in R was used to define DEGs between compared samples, using a threshold of fold change >1.5 and *P* < .05. The heat map was drawn using the ‘pheatmap’ package in R. eggNOG and TBtools were used for DEG enrichment analysis, and the ‘ggplot2’ package in R was used for drawing [39, 40].

### Whole-genome bisulfite sequencing and analysis

Genomic DNA was isolated from leaves of 30-day-old rTTT0 and rsTTT0 seedlings using the cetyl trimethyl ammonium bromide (CTAB) method [41]. Bisulfite treatment (EZ DNA Methylation Gold Kit, ZYMO Research), library construction (Accel-NGS Methyl-Seq DNA Library Kit, Swift Biosciences), and sequencing (Illumina Novaseq) were performed at the Novogene Bioinformatics Institute (Novogene, China). Fastp was used to trim reads with adapters and low-quality reads [42]. The remaining clean reads were mapped to the reference genome (*B. juncea* var. *tumida*, http://39.100.233.196:82/download_genome/Brassica_Genome_data/Braju_tum_V2.0) using Bismark [43]. The DNA methylation level (ML) for each C site was defined as ML(C) = reads (mC)/reads (mC + umC), where mC is C site with methylation and umC is C site without methylation. DMRs were identified using DSS software. In this study genes that overlapped DMRs in their gene body region (from TSS to TES) or promoter region (2 kb upstream from the TSS) were referred to as DMGs.

### Small RNA library construction for sequencing

Fourth leaves were collected from 30-day-old rTTT0 and rsTTT0 seedlings, and total RNA was extracted from them using TRIzol. Small RNA sequencing libraries were constructed using the NEBNext Multiplex Small RNA Library Prep Set for Illumina then sequenced on the Illumina HiSeq 2500 platform (Illumina) to produce 50-bp single-end reads at the Novogene Bioinformatics Institute (Novogene, China).

### MicroRNA identification and miRNA target predictions

Adapter sequences from small RNA libraries were trimmed, and low-quality reads were filtered out. Reads 18–30 nt in length were mapped to the *B. juncea* var. *tumida* reference genome (http://39.100.233.196:82/download_genome/Brassica_Genome_data/Braju_tum_V1.5/) using Bowtie2, with 0 mismatches [44]. Conserved miRNAs were identified using modified mirdeep2 software against mature miRNAs in miRBase (release20; http://www.mirbase.org/). Reads matching known structural RNAs from RepeatMasker and Rfam were removed. The remaining small RNAs were subjected to novel miRNA prediction by miREvo and mirdeep2 [45]. Read counts were standardized to transcripts per million (TPM) to compare miRNA levels across samples. The ‘DESeq2’ package in R was used to identify miRNAs with differential expression, and the criteria for a significant difference in expression was a fold change of >1.5 and a *P*-value of <0.05. A heat map was drawn using the ‘pheatmap’ package in R. Using TargetFinder’s default settings, miRNA targets were identified across the whole genome.

### Bisulfite sequencing

For BSP, genomic DNA was isolated from leaves of 30-day-old seedlings using the Plant Genomic DNA Kit (TIANGEN). Following the manufacturer’s instructions, an EZ DNA Methylation Gold Kit (ZYMO Research) was used to process the extracted DNA (500 ng). Using primers (Supplementary Data Table S1) that specifically targeted distinct *MIR* promoters and Taq DNA polymerase (Vazyme), the bisulfite-treated DNA was amplified by PCR in a 20-l reaction. The primers were designed using MethPrimer website (http://www.urogene.org/cgi-bin/methprimer2/MethPrimer.cgi). PCR conditions were 95°C for 5 minutes followed by 40 cycles of 95°C for 15 seconds, 45–50°C (adjusted according to primer pair and listed in Supplementary Data Table S3) for 15 seconds, and 72°C for 15 seconds, then 72°C for 10 minutes. The resulting PCR fragments were purified using the Wizard SV Gel and PCR Clean-Up System (Promega), cloned into the T5 vector (Transgen), and transformed into *Escherichia coli* DH5α competent cells. For each DMR locus, 12 independent clones were sequenced (ShangYa, China). Bisulfite DNA sequences and the levels of DNA methylation at the *MIR* promoter were analyzed using the online Kismeth tool (https://katahdin.girihlet.com/kismeth/revpage.pl) [46].

## Supplementary Material

Web_Material_uhad008

## Data Availability

All data are publicly available in NCBI under the accession number PRJNA878553.
