# Supplementary material for: The role of DNA methylation in the maintenance of phenotypic variation induced by grafting chimerism in Brassica
Source: Hortic Res. 2023 Jan 30;10(3):uhad008. doi: 10.1093/hr/uhad008 (PMC10028404; doi:10.1093/hr/uhad008)
Supplement: Web_Material_uhad008 [file web_material_uhad008.zip › supplementary tables_20221228.pdf]

Supplementary Table 1. Target genes of differentially expressed miRNAs between rTTT0 and rsTTT0

No.	miRNA	target gene	miRNA_log2FC	miRNA_Pvalue	gene_log2FC	gene_padj
1	aly-miR156e-3p	BjuVA01G31970	-0.67184	0.049541	0.657488838	0.018443964
1	aly-miR156e-3p	BjuVA10G13260	-0.67184	0.049541	1.280902086	0.011113433
1	aly-miR156e-3p	BjuVA02G28370	-0.67184	0.049541	5.400286405	0.020606097
1	aly-miR156e-3p	BjuVB06G05120	-0.67184	0.049541	0.622370493	0.005506834
1	aly-miR156e-3p	BjuVB06G40660	-0.67184	0.049541	7.046071231	0.0000262
2	aly-miR395i	BjuVA03G27140	-0.93223	0.031314	2.199491313	0.042190871
2	aly-miR395i	BjuVB04G15170	-0.93223	0.031314	0.611282798	0.04626797
2	aly-miR395i	BjuVA09G57340	-0.93223	0.031314	6.016305977	0.00000651
2	aly-miR395i	BjuVB06G41670	-0.93223	0.031314	5.454873422	0.02821668
2	aly-miR395i	BjuVB04G11650	-0.93223	0.031314	1.850358036	0.0000111
3	ath-miR172b-5p	BjuVA02G16690	0.75606	0.048838	-1.762514238	0.000000891
3	ath-miR172b-5p	BjuVA03G23620	0.75606	0.048838	-1.911518605	2.81E-11
3	ath-miR172b-5p	BjuVA05G07160	0.75606	0.048838	-2.525166984	0.008487767
3	ath-miR172b-5p	BjuVA08G26930	0.75606	0.048838	-2.097824345	0.0093215
3	ath-miR172b-5p	BjuVA04G24670	0.75606	0.048838	-1.112826751	2.66E-09
3	ath-miR172b-5p	BjuVB01G10990	0.75606	0.048838	-0.737412416	0.000467282
3	ath-miR172b-5p	BjuVB04G19310	0.75606	0.048838	-2.829191554	4.51E-08
3	ath-miR172b-5p	BjuVA09G32460	0.75606	0.048838	-0.78889744	0.000426813
3	ath-miR172b-5p	BjuVA05G13440	0.75606	0.048838	-0.737237664	0.032669508
4	ath-miR172c	BjuVA02G21850	0.78539	0.0032617	-0.632629821	0.018548928
4	ath-miR172c	BjuVA08G23100	0.78539	0.0032617	-0.773499053	0.011640677
4	ath-miR172c	BjuVA08G25940	0.78539	0.0032617	-4.033437222	0.001568382
4	ath-miR172c	BjuVA04G13560	0.78539	0.0032617	-2.692401834	0.00766167

No.	miRNA	target gene	miRNA_log2FC	miRNA_Pvalue	gene_log2FC	gene_padj
4	ath-miR172c	BjuVA04G15010	0.78539	0.0032617	-1.36976664	0.000110135
4	ath-miR172c	BjuVA08G32290	0.78539	0.0032617	-0.588735705	0.007755438
4	ath-miR172c	BjuVB06G27320	0.78539	0.0032617	-1.54493521	0.0000119
4	ath-miR172c	BjuVB05G12480	0.78539	0.0032617	-2.536142646	2.93E-16
4	ath-miR172c	BjuVB07G12150	0.78539	0.0032617	-0.792012034	0.029121064
4	ath-miR172c	BjuVB06G10110	0.78539	0.0032617	-0.991793849	0.000764694
4	ath-miR172c	BjuVB07G09950	0.78539	0.0032617	-1.703378044	0.003138823
5	ath-miR395a	BjuVA03G27140	-0.92723	0.029812	2.199491313	0.042190871
5	ath-miR395a	BjuVA09G57340	-0.92723	0.029812	6.016305977	0.00000651
5	ath-miR395a	BjuVB04G15170	-0.92723	0.029812	0.611282798	0.04626797
5	ath-miR395a	BjuVB06G41670	-0.92723	0.029812	5.454873422	0.02821668
6	ath-miR824-3p	BjuVA02G00210	0.8705	0.0043918	-2.112576472	0.011154032
6	ath-miR824-3p	BjuVA01G04230	0.8705	0.0043918	-0.856736732	0.045561567
6	ath-miR824-3p	BjuVA08G05990	0.8705	0.0043918	-1.155700931	0.0000171
6	ath-miR824-3p	BjuVA08G19330	0.8705	0.0043918	-0.608291126	0.018033482
6	ath-miR824-3p	BjuVB03G32300	0.8705	0.0043918	-0.791115354	0.003214739
6	ath-miR824-3p	BjuVB03G00090	0.8705	0.0043918	-2.804355343	0.016273499
6	ath-miR824-3p	BjuVA05G15490	0.8705	0.0043918	-0.740612848	0.033652271
6	ath-miR824-3p	BjuVA01G00810	0.8705	0.0043918	-1.026871018	0.037666417
6	ath-miR824-3p	BjuVB04G35800	0.8705	0.0043918	-1.289200942	0.003276
6	ath-miR824-3p	BjuVA05G04090	0.8705	0.0043918	-1.909002492	2.53E-12
6	ath-miR824-3p	BjuVA01G44000	0.8705	0.0043918	-1.475473436	0.0000623
6	ath-miR824-3p	BjuVB04G05820	0.8705	0.0043918	-2.325155909	9.48E-25
6	ath-miR824-3p	BjuVB05G30550	0.8705	0.0043918	-1.322831972	0.000658895
6	ath-miR824-3p	BjuVB08G36770	0.8705	0.0043918	-2.113058905	0.00000174

No.	miRNA	target gene	miRNA_log2FC	miRNA_Pvalue	gene_log2FC	gene_padj
6	ath-miR824-3p	BjuVA06G42170	0.8705	0.0043918	-0.892308606	0.021279311
6	ath-miR824-3p	BjuVB05G50610	0.8705	0.0043918	-1.543723916	0.0000042
6	ath-miR824-3p	BjuVB02G71030	0.8705	0.0043918	-0.750965035	0.042025451
7	ath-miR824-5p	BjuVB01G09560	1.0552	0.0009019	-1.782700997	0.00000583
7	ath-miR824-5p	BjuVA04G25850	1.0552	0.0009019	-2.223218705	1.25E-10
7	ath-miR824-5p	BjuVA03G01600	1.0552	0.0009019	-0.897245033	0.0000175
8	ath-miR845a	BjuVA07G19930	2.1384	3.134E-18	-1.145125117	0.017079286
9	bna-miR169m	BjuVB06G50220	0.70248	0.047262	-1.104300203	0.000011
10	bra-miR398-5p	BjuVB03G29220	-0.89162	0.000006127	1.742593436	0.003054384
10	bra-miR398-5p	BjuVA03G27140	-0.89162	0.000006127	2.199491313	0.042190871
11	bra-miR5711	BjuVB03G23800	0.68414	0.03786	-2.764982639	0.002757613
11	bra-miR5711	BjuVA06G25400	0.68414	0.03786	-2.357218003	0.012866814
11	bra-miR5711	BjuVB08G27290	0.68414	0.03786	-2.205814993	1.47E-08
12	bra-miR9552b-3p	BjuVA08G16470	-0.80446	3.5189E-06	0.630653371	0.000641876
12	bra-miR9552b-3p	BjuVA08G17120	-0.80446	3.5189E-06	0.725638932	0.000116588
13	fve-miR396b-3p	BjuVA05G12060	1.0066	0.020861	-0.700485185	0.010502495
13	fve-miR396b-3p	BjuVB07G02670	1.0066	0.020861	-1.222818906	0.00543362
13	fve-miR396b-3p	BjuVA03G04560	1.0066	0.020861	-0.70759487	0.009912841
13	fve-miR396b-3p	BjuVA09G48270	1.0066	0.020861	-1.397108195	0.000000698
13	fve-miR396b-3p	BjuVB02G73900	1.0066	0.020861	-1.125341411	0.00000135
13	fve-miR396b-3p	BjuVA03G63090	1.0066	0.020861	-2.303749809	2.73E-19
13	fve-miR396b-3p	BjuVB02G73900	1.0066	0.020861	-1.125341411	0.00000135
13	fve-miR396b-3p	BjuVA09G29730	1.0066	0.020861	-1.081787969	0.000227814
13	fve-miR396b-3p	BjuVB07G23110	1.0066	0.020861	-1.089300894	0.048592039
14	gma-miR396b-3p	BjuVA03G04560	0.92266	0.033396	-0.70759487	0.009912841

No.	miRNA	target gene	miRNA_log2FC	miRNA_Pvalue	gene_log2FC	gene_padj
14	gma-miR396b-3p	BjuVA05G12060	0.92266	0.033396	-0.700485185	0.010502495
14	gma-miR396b-3p	BjuVA09G29730	0.92266	0.033396	-1.081787969	0.000227814
14	gma-miR396b-3p	BjuVA08G23100	0.92266	0.033396	-0.773499053	0.011640677
14	gma-miR396b-3p	BjuVA03G63090	0.92266	0.033396	-2.303749809	2.73E-19
14	gma-miR396b-3p	BjuVB02G73900	0.92266	0.033396	-1.125341411	0.00000135
14	gma-miR396b-3p	BjuVA02G40920	0.92266	0.033396	-4.935980388	3.1E-34
14	gma-miR396b-3p	BjuVB03G16500	0.92266	0.033396	-2.003184771	2.19E-08
14	gma-miR396b-3p	BjuVB07G23110	0.92266	0.033396	-1.089300894	0.048592039
15	novel_106	BjuVB08G48510	2.0288	1.0521E-13	-1.114152482	0.00000238
16	novel_144	BjuVA02G13800	1.1043	0.01062	-2.563812905	0.00000721
16	novel_144	BjuVB01G21030	1.1043	0.01062	-1.241769515	0.013861247
16	novel_144	BjuVA03G26310	1.1043	0.01062	-0.823366029	0.00000606
16	novel_144	BjuVA09G42300	1.1043	0.01062	-0.687270928	0.000170816
16	novel_144	BjuVA04G14590	1.1043	0.01062	-1.180141717	0.013759764
16	novel_144	BjuVA07G21050	1.1043	0.01062	-1.777783638	0.000305247
16	novel_144	BjuVB08G33980	1.1043	0.01062	-1.943057285	0.0000926
16	novel_144	BjuVA03G63700	1.1043	0.01062	-0.898361743	0.000243972
16	novel_144	BjuVA07G21050	1.1043	0.01062	-1.777783638	0.000305247
16	novel_144	BjuVB06G50660	1.1043	0.01062	-2.127712392	0.000000687
16	novel_144	BjuVB07G16490	1.1043	0.01062	-1.046191456	0.000114957
17	novel_41	BjuVB06G34880	-2.7338	2.4532E-18	0.841151524	0.036612628
18	novel_53	BjuVA06G35480	0.75154	0.0048454	-1.885541223	6.52E-13
19	novel_57	BjuVB03G44030	1.836	2.0826E-08	-2.757048489	0.024524689
19	novel_57	BjuVB04G40370	1.836	2.0826E-08	-3.173054884	0.000044
19	novel_57	BjuVA03G28530	1.836	2.0826E-08	-0.808200214	0.049427499

No.	miRNA	target gene	miRNA_log2FC	miRNA_Pvalue	gene_log2FC	gene_padj
19	novel_57	BjuVA04G07890	1.836	2.0826E-08	-3.490638724	8.91E-11
20	novel_58	BjuVB02G23960	-1.1158	0.0054398	5.959817242	0.002131746
20	novel_58	BjuVB06G03000	-1.1158	0.0054398	1.680229584	0.005801768
20	novel_58	BjuVA07G22630	-1.1158	0.0054398	0.692966526	0.002489684
20	novel_58	BjuVA09G49280	-1.1158	0.0054398	1.232524788	0.000760507
21	novel_98	BjuVB02G35630	-2.0783	2.1619E-12	1.704448355	0.0000184
21	novel_98	BjuVA03G41680	-2.0783	2.1619E-12	1.241811367	0.002511264
22	osa-miR166d-5p	BjuVB03G44030	1.7041	5.8441E-11	-2.757048489	0.024524689
22	osa-miR166d-5p	BjuVB04G40370	1.7041	5.8441E-11	-3.173054884	0.000044
22	osa-miR166d-5p	BjuVB02G42440	1.7041	5.8441E-11	-2.299290301	1.38E-16
23	osa-miR408-3p	BjuVB02G57810	-0.62187	0.014028	0.866591073	0.019174801
23	osa-miR408-3p	BjuVA05G20650	-0.62187	0.014028	0.665231319	0.027465565
24	ppt-miR319a	BjuVA05G34510	-0.68035	0.049776	0.6373811	0.00159293
24	ppt-miR319a	BjuVA09G03160	-0.68035	0.049776	0.741335642	0.003076033
24	ppt-miR319a	BjuVB05G24250	-0.68035	0.049776	1.187105291	4.68E-11
25	pta-miR319	BjuVA05G34510	-0.68563	0.047306	0.6373811	0.00159293
25	pta-miR319	BjuVB04G50480	-0.68563	0.047306	0.724617594	0.0000417
25	pta-miR319	BjuVB05G24250	-0.68563	0.047306	1.187105291	4.68E-11
25	pta-miR319	BjuVB06G56860	-0.68563	0.047306	0.909211979	0.005681725
25	pta-miR319	BjuVB06G00760	-0.68563	0.047306	1.095199457	0.017743599
26	ptc-miR396e-3p	BjuVA03G04560	1.0358	0.017218	-0.70759487	0.009912841
26	ptc-miR396e-3p	BjuVA07G38240	1.0358	0.017218	-1.076988118	0.002393091
26	ptc-miR396e-3p	BjuVA03G63090	1.0358	0.017218	-2.303749809	2.73E-19
26	ptc-miR396e-3p	BjuVB02G73900	1.0358	0.017218	-1.125341411	0.00000135
26	ptc-miR396e-3p	BjuVA09G29730	1.0358	0.017218	-1.081787969	0.000227814

No.	miRNA	target gene	miRNA_log2FC	miRNA_Pvalue	gene_log2FC	gene_padj
26	ptc-miR396e-3p	BjuVA08G23100	1.0358	0.017218	-0.773499053	0.011640677
26	ptc-miR396e-3p	BjuVA06G19410	1.0358	0.017218	-1.219982068	0.021728049
26	ptc-miR396e-3p	BjuVA02G40920	1.0358	0.017218	-4.935980388	3.1E-34
26	ptc-miR396e-3p	BjuVB03G16500	1.0358	0.017218	-2.003184771	2.19E-08
26	ptc-miR396e-3p	BjuVA09G47460	1.0358	0.017218	-1.62732435	0.000000792
26	ptc-miR396e-3p	BjuVA01G16500	1.0358	0.017218	-2.492382932	1.91E-15
26	ptc-miR396e-3p	BjuVA08G26930	1.0358	0.017218	-2.097824345	0.0093215
26	ptc-miR396e-3p	BjuVA03G48520	1.0358	0.017218	-0.713768776	0.049740369
26	ptc-miR396e-3p	BjuVA03G06180	1.0358	0.017218	-1.115346512	0.001011327
26	ptc-miR396e-3p	BjuVB01G04900	1.0358	0.017218	-1.611742473	0.0000501
26	ptc-miR396e-3p	BjuVB08G47610	1.0358	0.017218	-0.967265809	0.000635186
26	ptc-miR396e-3p	BjuVB07G23110	1.0358	0.017218	-1.089300894	0.048592039
26	ptc-miR396e-3p	BjuVB08G29780	1.0358	0.017218	-1.959000865	0.045042647
27	stu-miR393-3p	BjuVB03G06800	0.9128	0.042873	-3.836989753	1.01E-08
27	stu-miR393-3p	BjuVB01G06710	0.9128	0.042873	-0.74333586	0.015343012
27	stu-miR393-3p	BjuVA03G18010	0.9128	0.042873	-1.185514512	0.038209547
28	tae-miR395b	BjuVA03G27140	-0.88339	0.041536	2.199491313	0.042190871
28	tae-miR395b	BjuVB04G15170	-0.88339	0.041536	0.611282798	0.04626797
28	tae-miR395b	BjuVB01G23090	-0.88339	0.041536	1.377747342	0.035930803
28	tae-miR395b	BjuVB02G01170	-0.88339	0.041536	7.621011528	0.000000268
29	zma-miR156k-5p	BjuVA04G31730	1.5963	0.00041055	-1.33805734	0.000372323
29	zma-miR156k-5p	BjuVA07G32060	1.5963	0.00041055	-1.63169767	0.000000142
29	zma-miR156k-5p	BjuVA10G12620	1.5963	0.00041055	-0.902562269	0.025165269
29	zma-miR156k-5p	BjuVA02G37620	1.5963	0.00041055	-1.110413131	0.00000161
29	zma-miR156k-5p	BjuVB04G11280	1.5963	0.00041055	-1.402637296	0.027109111

No.	miRNA	target gene	miRNA_log2FC	miRNA_Pvalue	gene_log2FC	gene_padj
29	zma-miR156k-5p	BjuVA09G57930	1.5963	0.00041055	-1.135654197	2.42E-10
29	zma-miR156k-5p	BjuVA06G06900	1.5963	0.00041055	-1.411913502	0.043230432
29	zma-miR156k-5p	BjuVA03G31060	1.5963	0.00041055	-1.791479316	6.97E-12
29	zma-miR156k-5p	BjuVA08G19330	1.5963	0.00041055	-0.608291126	0.018033482
29	zma-miR156k-5p	BjuVB01G21030	1.5963	0.00041055	-1.241769515	0.013861247
29	zma-miR156k-5p	BjuVB07G23070	1.5963	0.00041055	-1.283140787	0.002133939
29	zma-miR156k-5p	BjuVB05G13750	1.5963	0.00041055	-5.806459616	0.00437394
29	zma-miR156k-5p	BjuVB07G03240	1.5963	0.00041055	-0.705941586	0.034100854
29	zma-miR156k-5p	BjuVB06G32250	1.5963	0.00041055	-2.218266683	3.5E-10
29	zma-miR156k-5p	BjuVB05G01160	1.5963	0.00041055	-0.776169106	0.005833208
29	zma-miR156k-5p	BjuVB05G13680	1.5963	0.00041055	-1.700732207	0.004873238
29	zma-miR156k-5p	BjuVB06G32250	1.5963	0.00041055	-2.218266683	3.5E-10

Supplementary Table 2. Target genes of miRNAs whose expression levels were altered by graft chimera in rTTT0 and their biological functions

No.	miRNA	Up/Down	target mRNA	*Arabidopsis thaliana* homologous	description	mRNA_log2FC	mRNA_padj
1	ath-miR845a	Up	BjuVA07G19930	AT1G15040	Encodes a nitrogen regulated putative glutamine amidotransferase that represses shoot branching.	-1.14513	0.017079
2	novel_106	Up	BjuVB08G48510	AT3G49120	Class III peroxidase Perx34. Expressed in roots, leaves and stems. Located in the cell wall. Involved in cell elongation. Expression activated by light. May play a role in generating H_2O_2 during defense response. The mRNA is cell-to-cell mobile.	-1.11415	2.38E-06
3	osa-miR166d-5p	Up	BjuVB02G42440	AT5G06320	Encodes a protein whose sequence is similar to tobacco hairpin-induced gene (HIN1) and Arabidopsis non-race specific disease resistance gene (NDR1).	-2.29929	1.38E-16

No.	miRNA	Up/Down	target mRNA	*Arabidopsis thaliana* homologous	description	mRNA_log2FC	mRNA_padj
					Expression of this gene is induced by cucumber mosaic virus, spermine and Pseudomonas syringae pv. tomato DC3000. The gene product is localized to the plasma membrane.		
3	osa-miR166d-5p	Up	BjuVB03G44030	AT1G71450	Encodes a member of the DREB subfamily A-4 of ERF/AP2 transcription factor family. The protein contains one AP2 domain. There are 17 members in this subfamily including TINY. FUF1 appears to negatively regulate certain ethylene responsive EDF genes thereby negatively regulating flower senescence.	-2.75705	0.024525
3	osa-miR166d-5p	Up	BjuVB04G40370	AT1G30370	Encodes a mitochondria-localized class III phospholipase A1 that plays a role in seed viability.	-3.17305	4.40E-05

No.	miRNA	Up/Down	target mRNA	*Arabidopsis thaliana* homologous	description	mRNA_log2FC	mRNA_padj
4	novel_57	Up	BjuVA03G28530	AT4G10810	Hypothetical protein.	-0.8082	0.049427
4	novel_57	Up	BjuVB03G44030	AT1G71450	Encodes a member of the DREB subfamily A-4 of ERF/AP2 transcription factor family. The protein contains one AP2 domain. There are 17 members in this subfamily including TINY. FUF1 appears to negatively regulate certain ethylene responsive EDF genes thereby negatively regulating flower senescence.	-2.75705	0.024525
4	novel_57	Up	BjuVB04G40370	AT1G30370	Encodes a mitochondria-localized class III phospholipase A1 that plays a role in seed viability.	-3.17305	4.40E-05
4	novel_57	Up	BjuVA04G07890	AT4G14450	A member of a small family of proline/serine rich proteins of unknown function. It interacts with defense related MAP kinase MPK6. It's induced by PAMP elicitors. May play a	-3.49064	8.91E-11

No.	miRNA	Up/Down	target mRNA	*Arabidopsis thaliana* homologous	description	mRNA_log2FC	mRNA_padj
					role in response to pathogens.		
5	ath-miR824-5p	Up	BjuVA03G01600	AT5G04410	NAC family member, functions as a transcriptional activator, regulates flavonoid biosynthesis under high light. The mRNA is cell-to-cell mobile.	-0.89725	1.75E-05
5	ath-miR824-5p	Up	BjuVA04G25850	AT2G33580	Encodes a putative LysM-containing receptor-like kinase. LYK5 is a major chitin receptor and forms a chitin-induced complex with related kinase CERK1. Based on protein sequence alignment analysis, it was determined as a pseudo kinase due to a lack of the ATP-binding P-loop in the kinase domain.	-2.22322	1.25E-10
5	ath-miR824-5p	Up	BjuVB01G09560	AT2G33580	Encodes a putative LysM-containing receptor-like kinase. LYK5 is a major chitin receptor and forms a	-1.7827	5.83E-06

No.	miRNA	Up/Down	target mRNA	*Arabidopsis thaliana* homologous	description	mRNA_log2FC	mRNA_padj
					chitin-induced complex with related kinase CERK1. Based on protein sequence alignment analysis, it was determined as a pseudo kinase due to a lack of the ATP-binding P-loop in the kinase domain.		
5	ath-miR824-5p	Up	BjuVA03G01600	AT5G04410	NAC family member, functions as a transcriptional activator, regulates flavonoid biosynthesis under high light. ANAC078 module downregulates auxin level during leaf development (serration).		
6	ppt-miR319a	Down	BjuVA05G34510	AT3G15030	Arabidopsis thaliana TCP family transcription factor. Regulated by miR319. Involved in heterchronic regulation of leaf differentiation. Promotes hypocotyl elongation when	0.6373811	1.59E-03

No.	miRNA	Up/Down	target mRNA	*Arabidopsis thaliana* homologous	description	mRNA_log2FC	mRNA_padj
					hyperactivated. Enhance hypocotyl cell elongation by directly activating YUC5.		
7	pta-miR319	Down	BjuVA05G34510	AT3G15031	Arabidopsis thaliana TCP family transcription factor. Regulated by miR319. Involved in heterchronic regulation of leaf differentiation. Promotes hypocotyl elongation when hyperactivated. Enhance hypocotyl cell elongation by directly activating YUC6.	0.6373811	1.59E-03
8	novel_41	Down	BjuVB06G34880	AT1G15910	Belongs to a subgroup of SGS3-like proteins that act redundantly in RNA-directed DNA methylation.	0.841152	0.036613
9	novel_98	Down	BjuVA03G41680	AT3G21760	Encodes HYR1, a UDP glycosyltransferase (UGT). HYR1 glucosylates hypostatin, an inhibitor of cell expansion in vivo to form a bioactive glucoside.	1.241811	0.002511

No.	miRNA	Up/Down	target mRNA	*Arabidopsis thaliana* homologous	description	mRNA_log2FC	mRNA_padj
9	novel_98	Down	BjuVB02G35630	AT5G15310	Member of the R2R3 factor gene family; MIXTA-like transcription factor that controls trichome maturation and cuticle formation.	1.704448	1.84E-05
10	bra-miR9552b-3p	Down	BjuVA08G16470	AT4G35100	A member of the plasma membrane intrinsic protein PIP. functions as aquaporin. Salt-stress-inducible MIP.	0.630653	0.000642
10	bra-miR9552b-3p	Down	BjuVA08G17120	AT4G34200	Encodes a 3-phosphoglycerate dehydrogenase that is essential for embryo and pollen development.	0.725639	0.000117
11	bra-miR398-5p	Down	BjuVA03G27140	AT5G50020	DHHC-type zinc finger family protein.	2.199491	0.042191
11	bra-miR398-5p	Down	BjuVB03G29220	AT1G24530	Transducin/WD40 repeat-like superfamily protein.	1.742593	0.003054
12	ath-miR408-5p	Down	BjuVB01G36680	AT1G48650	DEA(D/H)-box RNA helicase family protein.	4.768372	1.58E-07
12	ath-miR408-5p	Down	BjuVB01G02150	AT2G44040	Dihydrodipicolinate reductase, bacterial/plant.	0.719365	0.004155

No.	miRNA	Up/Down	target mRNA	*Arabidopsis thaliana* homologous	description	mRNA_log2FC	mRNA_padj
12	ath-miR408-5p	Down	BjuVB07G11120	AT3G21460	Encodes a member of the CC-type glutaredoxin (ROXY) family that has been shown to interact with the transcription factor TGA2 and suppress ORA59 promoter activity.	4.55313	1.73E-20
13	aly-miR408-5p	Down	BjuVB01G36680	AT1G48650	DEA(D/H)-box RNA helicase family protein.	4.768372	1.58E-07
13	aly-miR408-5p	Down	BjuVB01G02150	AT2G44040	Dihydrodipicolinate reductase, bacterial/plant.	0.719365	0.004155
13	aly-miR408-5p	Down	BjuVB07G11120	AT3G21460	Encodes a member of the CC-type glutaredoxin (ROXY) family that has been shown to interact with the transcription factor TGA2 and suppress ORA59 promoter activity.	4.55313	1.73E-20

Supplementary Table 3. Primers used in analyses of cell type identification and bisulfite sequencing.

primer	sequence (5' to 3')	Tm (℃)
atpA F	GCTGCTTACAGGAGTTAGCC	55
atpA R	GTCCAATCGCTACATAGACA	
ARF10 F	TTTGTAGGAATATAGGGAGTTAGG	49
ARF10 R	CAAATTCCATAAAAACTTTACAATAACC	
IAA20 F	ATGTTGATATGTTGTAGGGAGTTTA	50
IAA20 R	CTTATATAAAAATTAATCCTTACCTTTTCT	
ROF1 F	GGGGAAGGTTATGTGAGTATTTTAA	50
ROF1 R	AACTTTTCAACCTACCTATAATCTTACAAC	
TPR2 F	TTGAAATTTTGAGAATAAAAAGGAT	47
TPR2 R	TAACTAAATAAACTAACTAATAAAACCCAT	
MIR319 F	TAAATAGGATTGTTTAAGTAAAATG	45
MIR319 R	AAAAAATTAAAATAAAATAAAATATAAAAT	
MIR824 F	TAGTTAAAAAAATTATAAAGAAGTTGGAAA	49
MIR824 R	AAAACACTAATTAAAAACCTATACTTTA	
