# Supplementary figures and images for: The role of DNA methylation in the maintenance of phenotypic variation induced by grafting chimerism in Brassica
Source: Hortic Res. 2023 Jan 30;10(3):uhad008. doi: 10.1093/hr/uhad008 (PMC10028404; doi:10.1093/hr/uhad008)

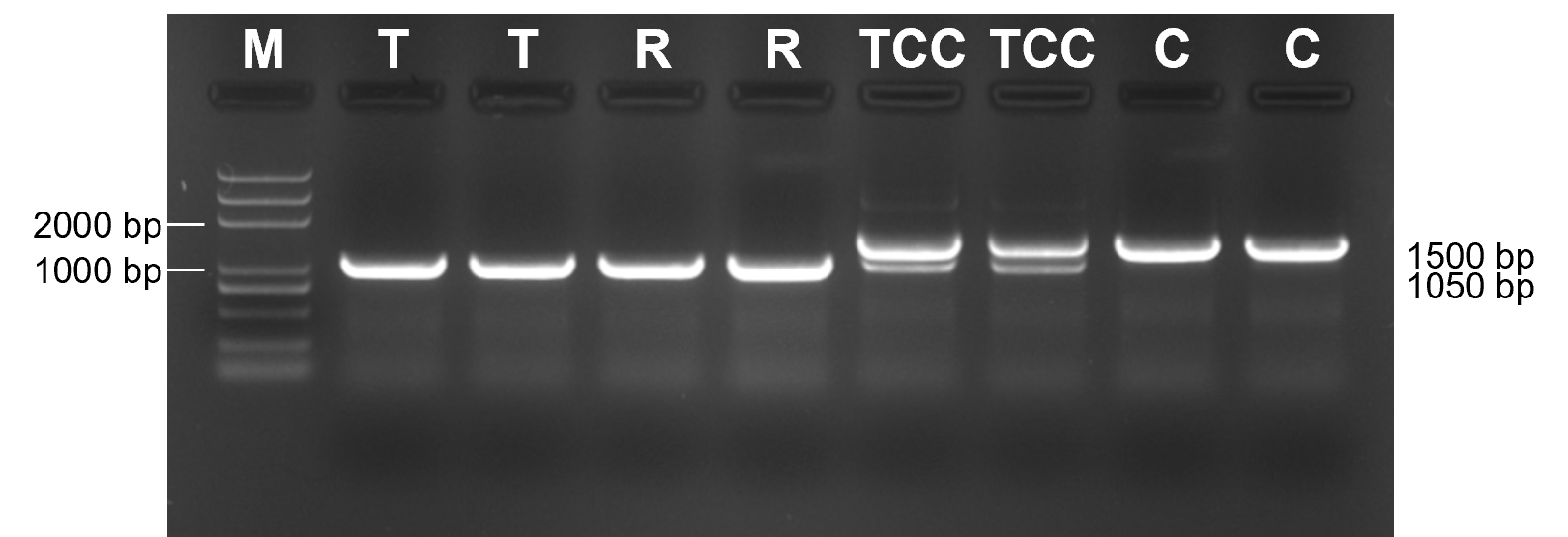

Supplement: Web_Material_uhad008 [file web_material_uhad008.zip › Figure S1.tif]

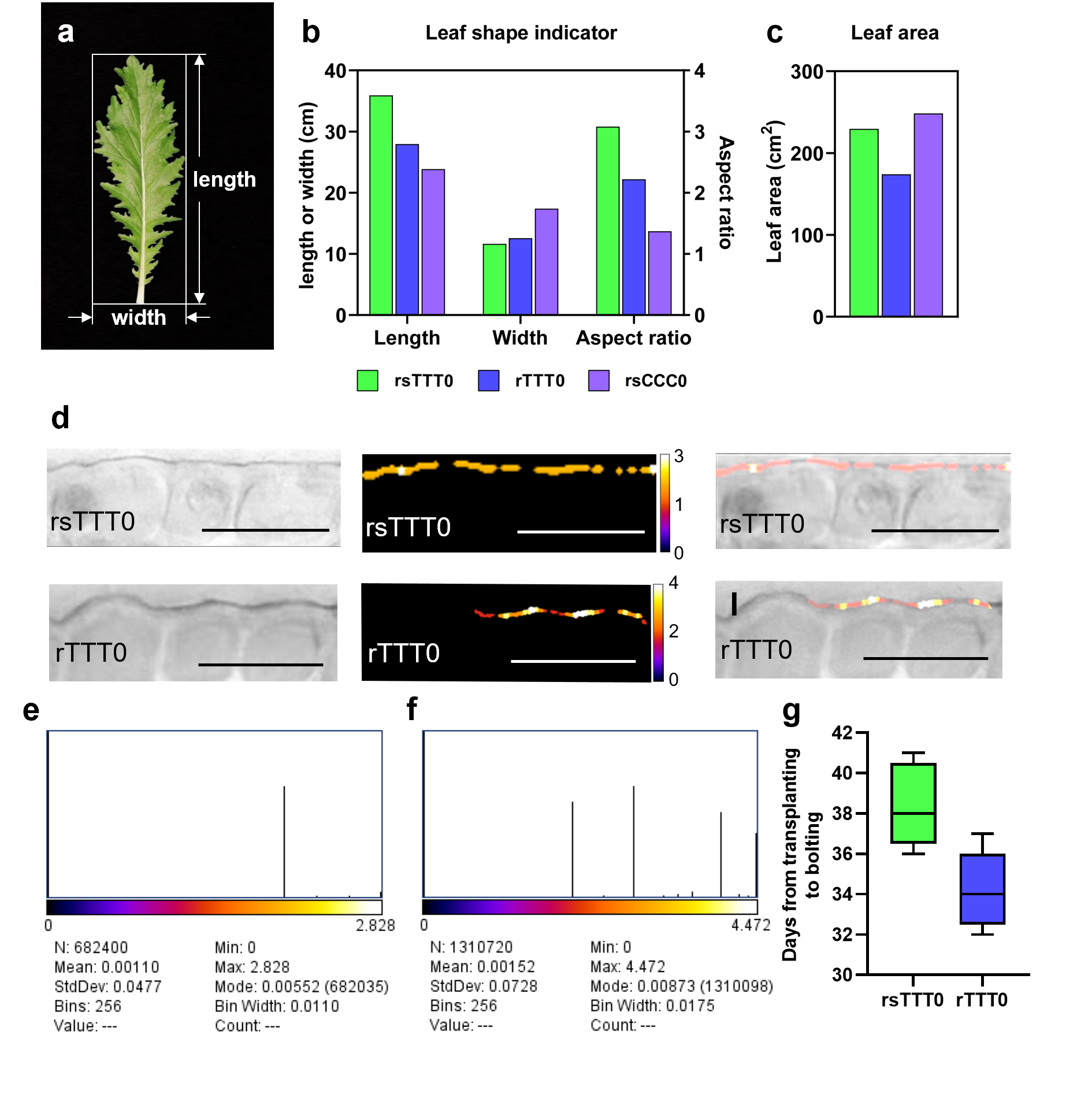

Supplement: Web_Material_uhad008 [file web_material_uhad008.zip › Figure S2.tif]

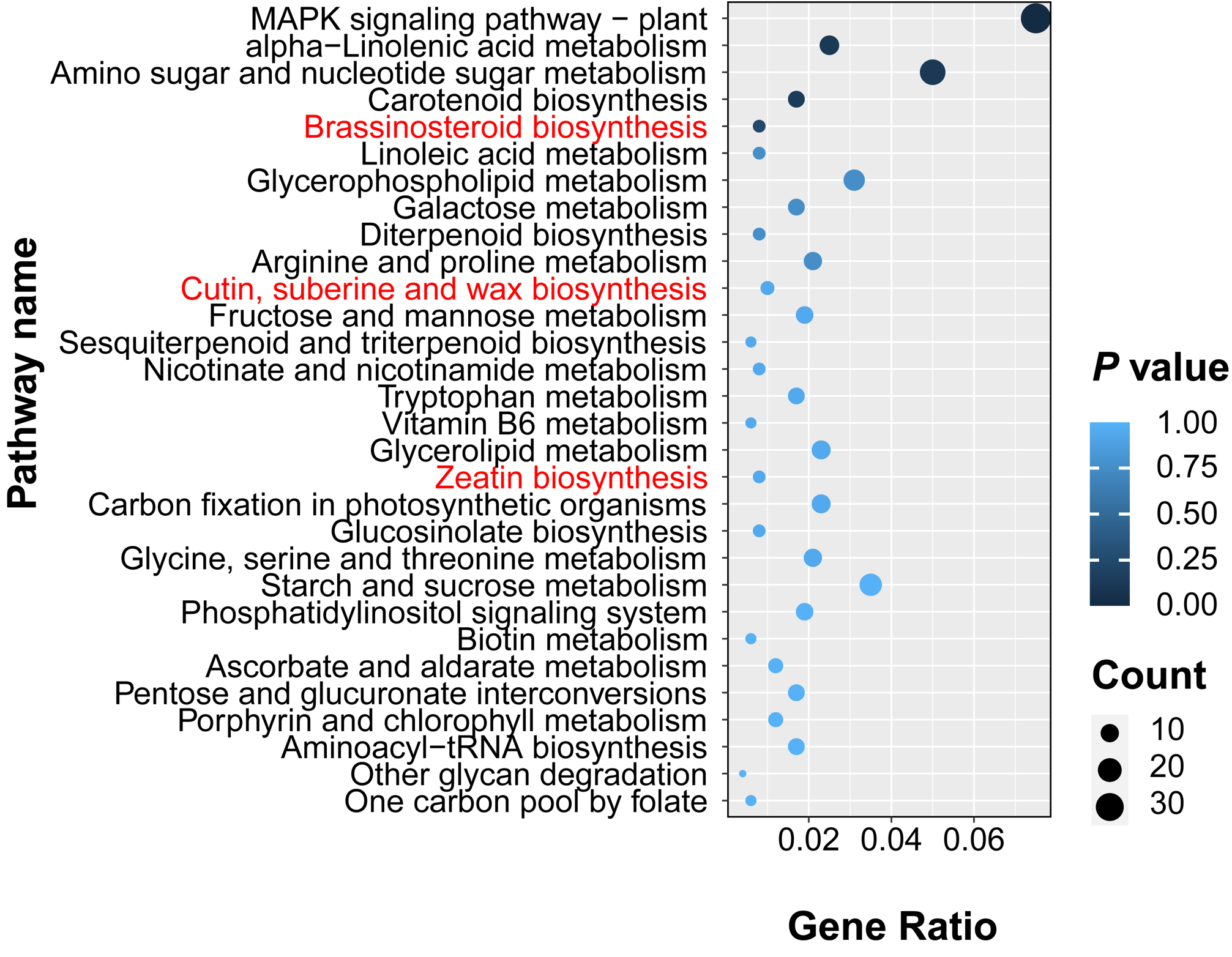

Supplement: Web_Material_uhad008 [file web_material_uhad008.zip › Figure S3.tif]
